# Editorial: Chlorophyll Fluorescence Imaging Analysis in Biotic and Abiotic Stress

**DOI:** 10.3389/fpls.2021.658500

**Published:** 2021-04-14

**Authors:** Michael Moustakas, Ángeles Calatayud, Lucia Guidi

**Affiliations:** ^1^Department of Botany, School of Biology, Aristotle University of Thessaloniki, Thessaloniki, Greece; ^2^Departamento de Horticultura, Centro de Citricultura y Producción Vegetal, Instituto Valenciano de Investigaciones Agrarias, Valencia, Spain; ^3^Department of Agriculture, Food and Environment, University of Pisa, Pisa, Italy

**Keywords:** photochemical efficiency, photosynthetic heterogeneity, reactive oxygen species, environmental stress, energy partitioning, oxidative stress, photosystem II, non-photochemical quenching

Chlorophyll *a* fluorescence that results from the absorbed light energy can be interpreted in terms of photosynthetic activity to obtain information about the state of photosynthetic apparatus and especially of photosystem II (PSII) (Krause and Weis, 1991; Murchie and Lawson, 2013). Measurements of chlorophyll *a* fluorescence have been extensively used to probe the function of the photosynthetic machinery and for screening different crops for plant tolerance to numerous stresses, and nutritional requirements (Guidi and Calatayud, 2014; Sperdouli and Moustakas, 2014; Kalaji et al., 2016; Bayçu et al., 2018). However, photosynthetic performance is not homogeneous at the leaf surface, which makes conventional chlorophyll fluorescence analysis non-representative of the physiological status of the whole leaf (Barbagallo et al., 2003; Moustakas et al., 2019a,b).

The development of chlorophyll fluorescence imaging instruments that are capable of identifying spatiotemporal heterogeneity of leaf photosynthetic performance offer new possibilities to understand the operation and regulation of photosynthesis at the whole leaf surface that cannot be identified through conventional chlorophyll fluorescence analysis (Calatayud et al., 2006; Chaerle et al., 2007; Sperdouli et al., 2019).

In the present work, we summarize the articles in this Research Topic that update the readers on the subject, and discuss current applications of chlorophyll fluorescence imaging analysis. Shimadzu et al., by combining gas-exchange and chlorophyll fluorescence measurements, demonstrated that whole irradiated plant promoted its photosynthetic induction, *via* improved stomatal opening, compared with individually irradiated leaf. By analyzing chlorophyll fluorescence data, Guo et al., revealed that gliotoxin (GT), a fungal secondary metabolite, affects both PSII electron transport at the acceptor side, and the reduction rate of PSI end electron acceptors' pool. They concluded that GT inserts in the plastoquinone Q_B_-site by replacing native plastoquinone, interrupting electron flow beyond plastoquinone Q_A_, and thus it may have the potential for being utilized as bioherbicide.

Bisphenol A (BPA), an intermediate chemical used for synthesizing plastic materials, has a spot-like mode of action on *Arabidopsis thaliana* leaves that was revealed by Adamakis et al., using chlorophyll fluorescence imaging analysis. They concluded that the necrotic death-like spots under BPA exposure could be due to reactive oxygen species (ROS) accumulation, while on the other hand the increased hydrogen peroxide (H_2_O_2_) generation played a role in the leaf response against BPA. ROS can activate the plant's defense mechanisms to cope with the oxidative stress and are essential for redox sensing, signaling, and regulation (Foyer, 2018; Moustaka et al., 2020; Adamakis et al., 2021a,b).

The ability to retain “a memory” or “stress imprint” of prior exposure to certain priming conditions, for a certain length of time, can make a plant more tolerant to future stress (Martinez-Medina et al., 2016). In this way, Wang et al. reported a study aimed to characterize the mechanism of heat acclimation memory in *Rhododendron hainanense*, a typical thermotolerant wild species. By transcriptomic and proteomic analysis it was evidenced that a lot of heat-responsive genes still maintained high protein abundance rather than transcript level after a recovery period of 2 days in heat-acclimated (37°C for 1 h) plants. From the transcriptome and proteome analyses, together with photochemical efficiency measurements, they were able to figure out the response patterns of chaperonins at transcript and protein levels in *R. hainanense*.

As mentioned above, chlorophyll *a* fluorescence imaging has been used to evaluate the effect of abiotic stress on photosynthesis. In this way, drought stress is one of the most important abiotic stresses in the world with a major effect the rapid accumulation of ROS that cause photoinhibition in PSII reaction centers (Sperdouli and Moustakas, 2012). Many researchers have studied the addition of external substances to cope with water stress in plants (Moustakas et al., 2011). In this sense, Huang et al. reported that leaf application of melatonin improved photosynthetic activity in maize seedlings through a higher photochemical activity mediated by the activation of antioxidative defense. The alleviation of water stress effects by melatonin application on leaves were higher when melatonin was applied in the roots compared with foliar spray indicating a melatonin signal from roots to leaves.

Chlorophyll fluorescence imaging has been utilized by Meng et al. to detect the virulence of 15 isolates of *Botrytis cinerea* on strawberry leaves grown under white, blue and red-light emitting diodes (LEDs). The maximum photochemical efficiency (F*v*/F*m*) ratio was strongly correlated with disease severity and it can represent a good indicator of the “gray mold” on strawberry leaves as has been previously shown (Guidi et al., 2007). In addition, Pérez-Bueno et al. recapitulate, in a review, photosynthetic responses to biotic stress, considering virus, bacteria, fungi, and pest, and evaluating their impact by chlorophyll fluorescence imaging analysis. In general terms, biotic stress impact on the leaf could be discriminated by spatial distribution of fluorescence quenching. On this matter, decrease in quenching fluorescence parameters and increase in non-photochemical quenching (NPQ) was observed in inoculated area, while in areas surrounding the infection, opposite behavior was detected. It was concluded that changes in chlorophyll fluorescence parameters can be observed prior to the development of visible symptoms, but there were not clear differences between healthy and infected leaf areas at late stages of the disease.

Finally, the Research Topic also illustrates the use of chlorophyll *a* fluorescence imaging not only to check plant performance under biotic and abiotic stresses, but also to the use of this technique to monitor the phytotoxic effect provoked by plant natural compounds (secondary plant metabolites). Sànchez-Moreiras et al. revealed that natural compounds can be highly phytotoxic at high doses or play a stimulant role at low doses. At phytotoxic level, the natural compounds reduced *F*_*v*_/*F*_*m*_, while the impact on non-photochemical quenching parameters was more variable, probably associated with the chemical structure of the compound or the dose applied.

Overall, from the significant articles presented it was concluded that photosynthetic performance is extremely heterogeneous at the leaf surface, especially under stress conditions, and that chlorophyll *a* fluorescence imaging constitutes a promising basis for investigating biotic and abiotic stress effects on plants. Contributions included in this e-book can be useful for scientists working on this topic, since recent advances in the subject were attractively presented and explained.

## Author Contributions

All authors listed have made a substantial, direct and intellectual contribution to the work, and approved it for publication.

## Conflict of Interest

The authors declare that the research was conducted in the absence of any commercial or financial relationships that could be construed as a potential conflict of interest.
